# Self correction fractional least mean square algorithm for application in digital beamforming

**DOI:** 10.1371/journal.pone.0304018

**Published:** 2024-06-21

**Authors:** Syed Asghar Ali Shah, Tariqullah Jan, Syed Muslim Shah, Muhammad Asif Zahoor Raja, Mohammad Haseeb Zafar, Sana Ul Haq

**Affiliations:** 1 Department of Electrical Engineering, University of Engineering and Technology Peshawar, Peshawar, Pakistan; 2 Department of Electrical Engineering, Capital University of Science and Technology Islamabad, Islamabad, Pakistan; 3 Graduate School of Engineering, Science and Technology, National Yunlin University of Science and Technology, Yunlin, Taiwan; 4 Cybersecurity and Information Networks Centre (CINC), Cardiff School of Technologies, Cardiff Metropolitan University, Cardiff, United Kingdom; 5 Department of Electronics, University of Peshawar, Peshawar, Pakistan; Guangdong University of Petrochemical Technology, CHINA

## Abstract

Fractional order algorithms demonstrate superior efficacy in signal processing while retaining the same level of implementation simplicity as traditional algorithms. The self-adjusting dual-stage fractional order least mean square algorithm, denoted as LFLMS, is developed to expedite convergence, improve precision, and incurring only a slight increase in computational complexity. The initial segment employs the least mean square (LMS), succeeded by the fractional LMS (FLMS) approach in the subsequent stage. The latter multiplies the LMS output, with a replica of the steering vector (*Ŕ*) of the intended signal. Mathematical convergence analysis and the mathematical derivation of the proposed approach are provided. Its weight adjustment integrates the conventional integer ordered gradient with a fractional-ordered. Its effectiveness is gauged through the minimization of mean square error (MSE), and thorough comparisons with alternative methods are conducted across various parameters in simulations. Simulation results underscore the superior performance of LFLMS. Notably, the convergence rate of LFLMS surpasses that of LMS by 59%, accompanied by a 49% improvement in MSE relative to LMS. So it is concluded that the LFLMS approach is a suitable choice for next generation wireless networks, including Internet of Things, 6G, radars and satellite communication.

## Introduction

**Fig 1:**
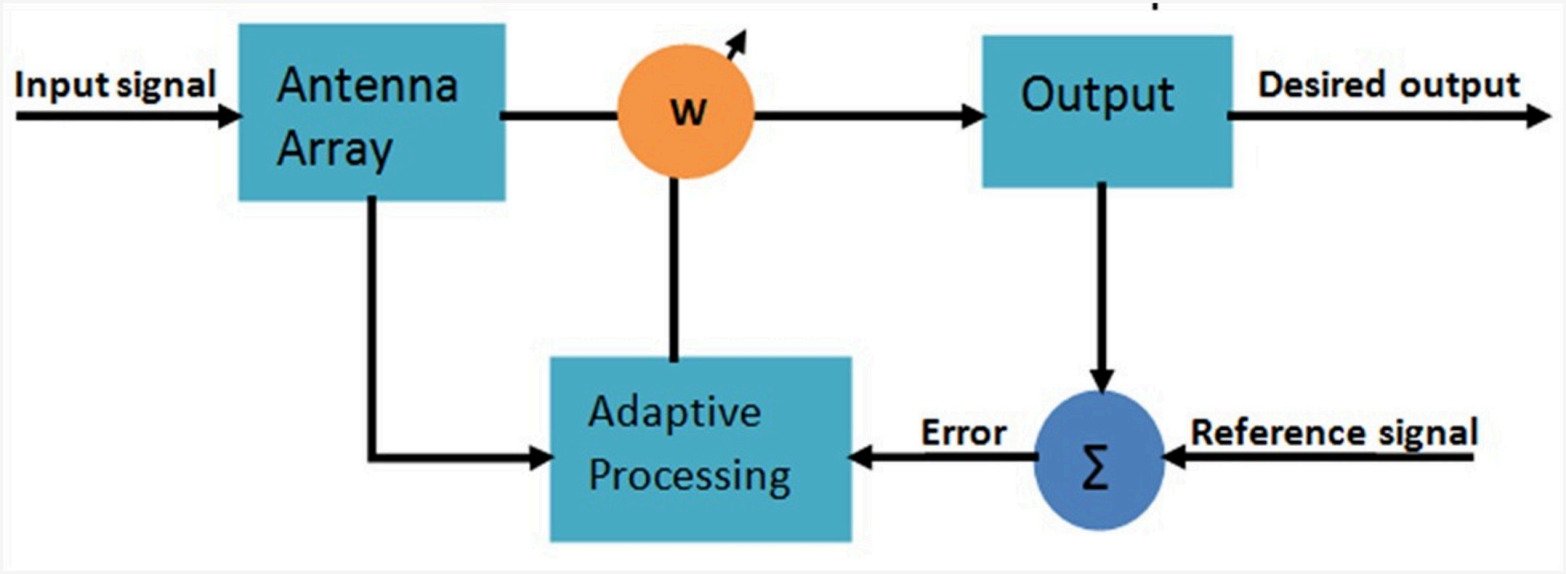
Adaptive beamforming block diagram.

Adaptive beamforming, a system within signal processing, leverages the spatial realm to enhance the performance of communications over wireless networks. It extends accessibility as well as boosts transmission speeds, especially in environments featuring multiple propagation paths. It has extensive applications in wide range of systems that includes the internet of things (IoT), communication systems, navigation, tracking systems, ranging and sonar [1–4]. Furthermore it is quickly evolving and has applications in other domains, such as ultrasound imaging [5], 5G technology [6], underwater communication [7] and network systems [8]. Accurately estimating the arrival angle is a crucial first step in beamforming [9,10]. This estimation process is very essential as it provides the basis for effective beamforming techniques utilized in various applications. Then it proceeds by using beamforming approaches [11] to generate a precise, focused beam in the desired direction, while effectively cancelling out the signals arriving from undesirable directions. The system is able to respond to variations in the environment quickly and effectively, because of its adaptability. This flexibility of the system is particularly useful in complex and varying communication situations. Fig 1 shows the general schematic design of adaptive beamforming. In these systems different algorithms are employed, each having its own method for updating their weight vectors. Among these the recursive least square (RLS) [12] and the constant modulus algorithm (CMA) [13] are well known. The LMS [14] is the popular choice because of its simplicity, good tracking capability and robustness to the variations. However there is a weakness of LMS, its relatively slow convergence speed. This has prompted the development of several modifications to LMS to enable faster convergence. The normalized least mean square (NLMS) [15] was introduced as a variant which exhibits improved performance, it also face issues in cases of smaller signal-to-noise ratio (SNR). The other modification is the variable step size least mean square (VSS-LMS) [16], which enhances convergence speed at the cost of computational complexity. Moreover alternative algorithms like the recursive least square-least mean square (RLMS) [17], least mean square-least mean square (LLMS) [18] and Kalman-based parallel RLMS algorithm [19] were introduced. These alternatives have shown enhanced convergence and stability performance compared to LMS with increased computational complexity. The LLMS algorithm constitutes of two concatenated LMS sections. Another double-stage LLMS variant [20] was formulated for concentric circular arrays. In order to address the concerns about complexity and to facilitate practical hardware implementation a Parallel LMS algorithm [21] was introduced. A scheme is still required that maintains a balance among convergence speed, mean square error (MSE), and computational cost. The field of fractional calculus has witnessed a surge in importance, primarily due to its diverse applications across various engineering disciplines. Systems based on FC exhibited notable advancements in numerous domains, ranging from equalization [22] to recommender systems [23]. In fields like noise control [24], line echo control [25], and beamforming [26], fractional variants have proven highly effective. In a comprehensive study [27], it was revealed that fractional order processing consistently outperforms conventional algorithms in various signal processing applications. Notably, the fractional LMS algorithm has found applications in power signal modeling [28] and system identification [29]. Furthermore, fractional calculus has made significant inroads into the domain of wireless sensor networks [30] and pulse arrival time detection in electrocardiography (ECG) signals [31]. Collectively, algorithms grounded in fractional processing consistently demonstrate superior performance when compared to their traditional counterparts and can be candidates for application in modern engineering disciplines such as control, instrumentation, and IoT, based future generations of communication systems.

### Contributions

In current research work, authors introduce a novel approach to adaptive beamforming that integrates first order as well as fractional order derivatives in weight adaptation. This two-stage algorithm, processes the signals received at array elements to assess the overall error and output, primarily through the MSE metric. The algorithm is mathematically derived along with its convergence analysis. The addition of non linear element arising from fractional derivatives modifies the autocorrelation matrix, reducing the dispersion of eigenvalues and improving convergence rates and steady state performance, as well as for substantial step sizes. In different scenarios to gauge its efficacy, the proposed algorithm is subjected to a comparative analysis against the FLMS [32] and its classical counterpart, utilizing convergence learning curves, scatter plots, and beampattern accuracy as assessment metrics. The results from this study unequivocally demonstrate the superiority of the proposed approach.

### Organization

The structure of the subsequent sections involves the introduction of a system model for the LFLMS approach and presenting an analysis of the FLMS algorithm’s convergence in Section 2. Section 3 highlights the outcomes of the simulations and provides a detailed analysis of the efficiency of the LFLMS adaptive beamforming system. Moreover, in Section 4, we bring the paper to a conclusion, with a summary of the key findings and recommendations for further research in the field.

## Proposed Model (LFLMS)

**Fig 2:**
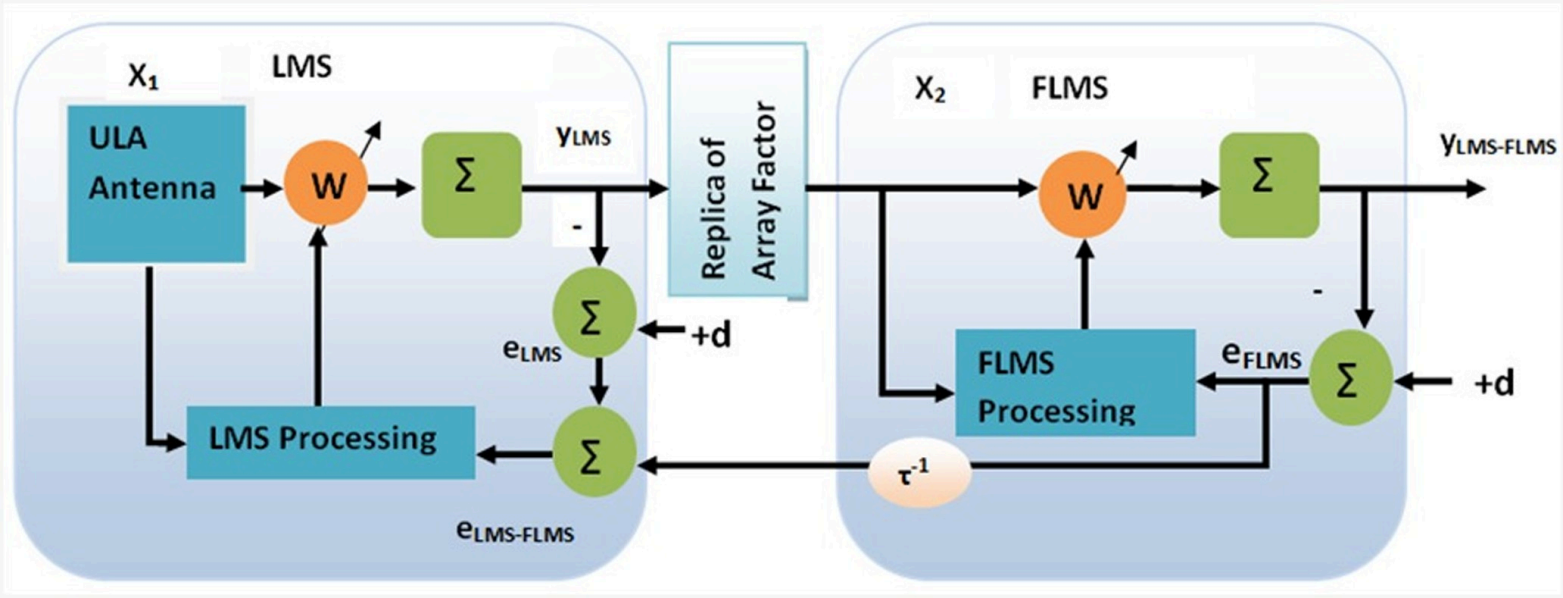
Proposed model block diagram.

The proposed adaptive array beamforming algorithm shown in Fig 2 comprises two distinct segments. Its initial segment employs the LMS algorithm, and the subsequent section exploits the fractional LMS approach. The replica of the steering vector (*Ŕ*) of the intended signal alienates segments of the proposed approach. This replica of the steering vector acts as a spatial filter. In accordance with the algorithm, the elements of the array are evenly spaced by half of the signal wavelength and are subject to interference from both desired and unwanted signals from *M* sources. The AOA for both the intended and interferer signals is presumed to be known; in contrast, it is calculated through an appropriate estimation algorithm [9,33–35]. The signals received by the array elements serve as the algorithm’s input. Weight coefficients are applied to the received signals to obtain intermediate output (*y*_*L*_). The discrepancy of *d* and *y*_*L*_ is the error signal(*e*_L_). Subsequently, the weights are adapted through an optimization process based on the error signal, facilitating the algorithm to converge by minimizing *e*_L_. MSE minimization is employed as a performance metric because of its mathematical tractability. The two sections are coordinated so that the output of the first segment of the algorithm is utilized as input in the subsequent segment. In order to draw a filtered signal, the input signal (*y*_*L*_) is subjected to multiplication by *Ŕ*, which acts as a spatial filter for the desired signal. The disparity between the second part’s output and reference signal yields (*e*_F_) is employed to adapt the weights of the second section FLMS and is also returned to the first part with one sample delay to determine the overall error(*e*_*LFLMS*_) of the suggested scheme, resulting in weight adjustment of the first part. This intricate interaction makes certain that the algorithm as a whole converges when each section converge. To avoid numerical errors when calculating the fractional power of a weight, transformation including the signum function and absolute value of the weight would be required. Additionally, the model is presumed to operate in a stationary environment. The incoming signals have a zero mean, are uncorrelated, and are characterized by statistical independence.

### Mathematical model of LFLMS

This configuration uses a transposed vector to represent the signal vector, which is comprised of signals received at array elements:

$$x={\left[x_{1},x_{2},x_{3},\ldots x_{M}\right]}^{T}$$ (1)

At instant *t*, the vector representing the array is articulated as:

$$x=S_{d}(t)a\left(\theta_{d}\right)+S_{i}(t)a\left(\theta_{i}\right)+b(t)$$ (2)

Here *θ*_*d*_ is the angle of arrival of the intended arriving signal *S*_*d*_ and *θ*_*i*_ is that of interfering signal *S*_i_. Steering vectors of desired and interfering signals are represented by ***a***(*θ*_*d*_) and ***a***(*θ*_*i*_). Whereas *b*(*t*) is white Gaussian noise. The input signal ***x*** is weighted by applying *h*_*L*_, to obtain the first segment’s output:

$$y_{L}=h_{L}^{H}x$$ (3)

*y*_*L*_(***x***_1_), is an estimate of the intended signal *S*_*d*_. Applying *Ŕ* to the output of the first section extracts the desired component ***x***_2_ out of the signal impinging from the desired angle. It is fed into the algorithm’s subsequent part, which further filters out noise and interference exploiting the fractional LMS algorithm.


$$x_{2}=Ŕx_{1}$$ (4)

The result of the fractional LMS, denoted as *y*_*F*_ is derived by applying weights to ***x***_2_ i.e.*y*_F_ = h^H^_FL_***x***_2_. By putting $x_{2}=Ŕx_{1}$ we get:

$$y_{F}=h_{F}^{H}Ŕx_{1}$$ (5)

Weights vectors *h*_*F*_ and *h*_*L*_ of the FLMS and LMS sections respectively, are updated by the following equations:

$$h_{F}(t+1)=h_{F}(t)+\mu e_{F}(t)x_{2}(t)+\mu e_{F}(t)x_{2}(t)⊙\frac{1}{\Gamma (2-\beta )}h^{1-\beta }$$ (6)

Weight vector of LMS is updated using following equation:

$$h_{L}(t+1)=h_{L}(t)+\mu e_{L}(t)x_{1}(t)$$ (7)

The error signals *e*_*L*_ and *e*_*F*_ correspond to the LMS and fractional LMS segments, accordingly. The size of step (*μ*) controls its rate, at which the weights are updated, and their values can have a significant impact on the convergence rate and stability. In the FLMS algorithm, *β* and *Γ* denotes the fractional order and gamma function, respectively. Here *d* = *d*_1_ = *d*_2_ represent the reference signals and *e*_*L*_ is the error signal:

$$e_{L}(t)=d_{l}(t)-h_{L}^{H}(t)x_{1}(t)$$ (8)

The error signal for FLMS section is obtained using the given equation:

$$e_{F}(t)=d_{2}(t)-h_{F}^{H}(t)x_{2}(t)$$ (9)

The following is the weight update *h*_*F*_(t + 1) equation, which gives the overall optimum weight of the algorithm:

$$h_{LFLMS}(t+1)=h_{LFLMS}(t)+\mu e_{LFLMS}(t)x_{2}(t)$$ (10)

For the LFLMS algorithm, the overall error is typically defined as a mathematical expression:

$$e_{LFLMS}(t)=e_{L}(t)-e_{F}(t-1)$$ (11)

Eq (6) can also be expressed as:

$$h_{F}(t+1)=h_{F}(t)+\mu e_{F}(t)Ŕh_{L}^{H}(t)x(t)+\mu e_{F}(t)Ŕh_{L}^{H}(t)x_{1}(t)⊙\frac{1}{\Gamma (2-\beta )}h_{F}^{1-\beta }$$ (12)

The mean squared error minimization function of the LFLMS algorithm is:

$$\xi (t)=E\left[{\left|e_{LFLMS}(t)\right|}^{2}\right]=E\left[{\left|e_{L}(t)-e_{F}(t-1)\right|}^{2}\right]$$ (13)

$\text{Let}\;D(\text{t})=d(\text{t})-e_{\text{F}}(\text{t}-1)\;\text{and}\;Q(\text{t})=\text{E}\left[x_{1}(\text{t})x_{1}^{\text{H}}(\text{t})\right],$ then Eq (13) can be written using Eqs (8) and (9):

$$\xi (t)=E\left[{\left|d_{1}(t)-h_{L}^{H}(t)x_{1}(t)-e_{F}(t-1)\right|}^{2}\right]$$ (14)

By putting $D(t)=d(t)-e_{F}(t-1)$ it transforms into:

$$\xi (t)=E\left[{\left|D(t)-h_{L}^{H}(t)x_{1}(t)\right|}^{2}\right]$$ (15)

After mathematical rearrangement, it results in:

$$\begin{array}{c} \xi (t)=E\left[|D(t){|}^{2}\right]+E\left[h_{L}^{H}(t)x_{1}(t)x_{1}^{H}(t)h_{L}(t)\right]-E\left[D(t)x_{1}^{H}(t)h_{L}(t)\right.\\ \left.+D^{*}(t)h_{L}^{H}(t)x_{1}(t)\right] \end{array}$$ (16)

By putting $Q(t)=\text{E}\left[x_{1}(t)x_{1}^{H}(t)\right]$ Eq (16) becomes:

$$\xi (t)=E\left[|D(t){|}^{2}\right]+h_{L}^{H}(t)Q(t)h_{L}(t)-E\left[D(t)x_{1}^{H}(t)h_{L}(t)+D^{*}(t)h_{L}^{H}(t)x_{1}(t)\right]$$ (17)

Now

$$E\left[|D(t){|}^{2}\right]=E\left[{\left|d_{1}(t)-e_{F}(t-1)\right|}^{2}\right]=E\left[{\left|d_{1}(t)\right|}^{2}\right]+E{\left[\left|e_{F}(t-1)\right|\right]}^{2}-E\left[d_{1}(t)e_{F}^{*}(t-1)+d_{1}^{*}(t)e_{F}(t-1)\right]$$ (18)

The final two terms in the equation become zero because they are uncorrelated and have a zero mean:

$$E\left[|D(t){|}^{2}\right]=E\left[{\left|d_{1}(t)\right|}^{2}\right]+E{\left[\left|e_{F}(t-1)\right|\right]}^{2}$$ (19)

Now consider the term $\text{E}{\left[\left|e_{\text{F}}(t-1)\right|\right]}^{2}$:

$$E{\left[\left|e_{F}(t-1)\right|\right]}^{2}=E\left[{\left|d_{2}(t-1)-y_{LFLMS}(t-1)\right|}^{2}\right]=E\left[{\left|d_{2}(t-1)\right|}^{2}\right]+E\left[{\left|y_{LFLMS}(t-1)\right|}^{2}\right]-E\left[d_{2}^{*}(t-1)y_{LFLMS}(t-1)+d_{2}(t-1)y_{LFLMS}^{*}(t-1)\right]$$ (20)

Since *d*_1_(t) = *d*_2_(t) and *y*_*LFLMS*_ is:

$$y_{LFLMS}(t)=h_{F}^{H}(t)x_{2}(n)=h_{F}^{H}(t)Ŕh_{L}^{H}(t)x_{1}(t)$$ (21)

Eq (20) may be written as:

$$E{\left[\left|e_{F}(t-1)\right|\right]}^{2}=E\left[{\left|d_{2}(t-1)\right|}^{2}\right]+E\left[h_{F}^{H}(t-1)Ŕh_{L}^{H}(t-1)X_{1}(t-1)x_{2}^{H}(t-1)h_{L}^{H}(t-1)Ŕh_{F}^{H}(t-1)\right]$$

$$-E\left[d_{2}^{*}(t-1)h_{F}^{H}(t-1)Ŕh_{1}^{H}(t-1)x_{1}(t-1)+d_{2}(t-1)h_{F}^{H}(t-1)Ŕh_{L}^{H}(t-1)x_{1}(t-1)\right]$$ (22)

By putting $h_{LFLMS}^{H}(t-1)=h_{F}^{H}(t-1)Ŕh_{L}^{H}(t-1)$ and $h_{LFLMS}(t-1)=h_{L}^{H}(t-1)Ŕh_{F}^{H}(t-1)$ it becomes:

$$E{\left[\left|e_{F}(t-1)\right|\right]}^{2}=E\left[{\left|d_{2}(t-1)\right|}^{2}\right]+h_{LFLMS}^{H}(t-1)E\left[x_{1}(t-1)x_{2}^{H}(t-1)\right]h_{LFLMS}(t-1)-h_{LFLMS}E\left[d_{2}^{*}(t-1)\right.$$

$$\left.\left.x_{1}(t-1)\right]+h_{LFLMS}^{H}(t-1)E\left[x_{1}(t-1)d_{2}^{*}(t-1)\right)\right]$$ (23)

By substituting $Q(t-1)=\text{E}\left[x_{1}(t-1)x_{1}^{H}(t-1)\right]$ and $\text{z}(t)=\text{E}\left[x_{1}(t)d_{2}^{*}(t)\right]$ we get:

$$E{\left[\left|e_{F}(t-1)\right|\right]}^{2}=E\left[{\left|d_{2}(t-1)\right|}^{2}\right]+h_{LFLMS}^{H}(t-1)Q(t-1)h_{LFLMS}(t-1)-h_{LFLMS}^{H}(t-1)z(t-1)$$

$$-z^{\text{H}}(t-1)h_{LFLMS}(t-1)$$ (24)

Here $z(t)=\text{E}\left[x_{1}(t)d_{2}^{*}(t)\right]$ is the cross correlation vector. Eq (19) now becomes:

$$E\left[|D(t){|}^{2}\right]=E\left[E\left[{\left|d_{1}(t)\right|}^{2}\right]+E\left[{\left|d_{2}(t-1)\right|}^{2}\right]-h_{LFLMS}^{H}(t-1)z(t-1)-z^{H}(t-1)h_{LFLMS}(t-1)\right.$$

$$\left.+h_{LFLMS}^{H}(t-1)Q(t-1)h_{LFLMS}(t-1)\right]$$ (25)

Given *d*_1_(*t*) = *d*_2_(*t*), consider the last term of Eq (17) that is:

$$E\left[D(t)x_{2}^{H}(t)h_{L}(t)+D^{*}(t)h_{L}^{H}(t)x_{1}(t)\right]=z^{H}(t)h_{L}(t)+h_{L}^{H}(t)z(t)$$ (26)

By putting Eqs (26) and (17):

$$\begin{array}{c} \xi (t)=E\left[{\left|d_{2}(t-1)\right|}^{2}\right]+E\left[{\left|d_{2}(t-1)\right|}^{2}\right]+h_{LFLMS}^{H}(t-1)Q(t-1)h_{LFLMS}(t-1)-z^{H}(t-1)h_{L}(t)-h_{LFLMS}^{H}(t-1)\\ z(t-1)-z^{H}(t-1)h_{LFLMS}(t-1)-h_{L}^{H}(t)z(t)+h_{L}^{H}(t)Q(t)h_{L}(t) \end{array}$$ (27)

Differentiating Eq (27) w. r. t.***h***_*L*_^*H*^(*t*) and setting it to zero for optimal weights:

$$\xi (t)=0+0+0-z(t)-z(t)+2Q(t)h_{L}(t)=0$$

$$-2z(t)+2Q(t)h_{opt1}(t)=0$$

Or $Q(t)h_{opt1}(t)=z(t)$

$$Orh_{opt1}(t)=Q{\left(t\right)}^{-1}z(t)$$ (28)

It is a matrix representation of the Weiner-Hoff equation. For minimum MSE, put Eqs (28) and (27):

$$\xi_{\min}(t)=E\left[{\left|d_{2}(t-1)\right|}^{2}\right]+E\left[{\left|d_{2}(t-1)\right|}^{2}\right]-z^{H}(t)h_{opt}(t)-z^{H}(t-1)h_{LFLMS}^{H}(t-1)+h_{LFLMS}^{H}(t-1)$$

$$z(t-1)\left\{Ŕh_{L}(t-1)-1\right\}$$ (29)

By putting in Eq (17):

$$\xi (t)=\xi_{min}(t)+{\left(h_{L}-h_{opt1}\right)}^{H}Q\left(h_{L}-h_{opt1}\right)$$

where $v_{1}=\left(h_{L}-h_{opt1}\right)$

$$\xi (t)=\xi_{min}+v_{1}^{H}Qv_{1}$$ (30)

The gradient of Eq (30) w. r. t. $v_{1}^{H}$ is:

$$\nabla \xi (t)=Qv_{1}$$ (31)

Since

$$Q=q_{1}\wedge_{1}q_{2}[\text{EVD}]$$

For steepest descent:

$$h_{L}(t-1)=h_{L}(t)+\mu \nabla \xi (t)$$ (32)

Correspondingly

$$v_{1}(t-1)=v_{1}(t)-\mu Qv_{1}(t)$$ (33)

Alternatively Eq (19) can be written as:

$$v_{1}(t)=\left(q_{1}q_{1}^{H}-\mu q_{1}\wedge_{1}q_{1}^{H}\right)v_{1}(t-1)$$ (34)

After simplification it may be written as:

$$v_{1}(t)=q_{1}{\left(I-\mu \wedge_{1}\right)}^{j}q_{1}^{H}v_{1}(0)$$ (35)

Table 1 summarizes the proposed scheme.

**Table 1:** Summary of algorithm.

**Initialize** ***x***_1_: received signal, ***x***_2_: input signal to FLMS section, *d*_1_ = *d*_2_: reference signal, *μ*:step size, *Ŕ*:replica of steering vector of desired signal, Initially set error signal and weight vectors to zero
$e_{L}(t)=d_{l}(t)-h_{L}^{H}(t)X_{1}(t)$
$e_{F}(t)=d_{2}(t)-h_{F}^{H}(t)X_{2}(t)$ $e_{LFLMS}(t)=e_{L}(t)-e_{F}(t-1)$
$h_{L}(t+1)=h_{L}(t)+\mu e_{L}(t)X_{1}(t)$
$h_{F}(t+1)=h_{F}(t)+\mu e_{F}(t)X_{2}(t)+\mu e_{F}(t)X_{2}(t)⊙\frac{1}{\Gamma (2-\propto )}h^{1-\beta }$
$y_{L}=h_{L}^{H}X_{1}$
$y_{F}=h_{F}^{H}X_{2}$
$X_{2}=ŔX_{1}$
$y_{LFLMS}(t)=h_{F}^{H}(t)X_{2}(t)=h_{F}^{H}(t)Ŕh_{L}^{H}(t)X_{1}(t)$
$h_{LFLMS}(t+1)=h_{LFLMS}(t)+\mu e_{LFLMS}(n)X_{2}(t)$

### Fractional LMS

The function *J*(*t*) of LMS involves the minimization of the MSE, which serves as a fundamental criterion for optimization. The weight adaptation relation is derived through an iterative method that involves the differentiation of *J*(*t*). This iterative process allows for the continuous refinement of the weight vector as it strives to minimize *J*(*t*) over successive iterations. The cost function is:

$$J(t)=E\left[|e(t){|}^{2}\right]=E\left[{\left\{d(t)-y(t)\right\}}^{2}\right]$$ (36)

After algebraic manipulation, it takes form:

$$J(t)=d^{2}(t)+h^{H}x(t)x^{H}h(t)-2d(t)h^{H}x(t)$$ (37)

By calculating the gradients of *J*(*t*) and subsequently adjusting the weights accordingly, the LMS algorithm progressively converges towards the optimal weight configuration:

$$h(t+1)=h(t)+\mu \frac{\partial J(t)}{\partial h}$$ (38)

Here ***h*** represents the vector of weight coefficients. This variant of the LMS algorithm incorporates the integer and fractional order corrective terms in weight adaptation. The fractional update term is added to Eq (38) and rephrased as:

$$h(t+1)=h(t)-\left[\frac{\mu \partial J(t)}{2\partial h}+\frac{\mu \partial^{\beta }J(t)}{2\partial^{\beta }h}\right]$$ (39)

Here *β* represents the fractional order having a value between 0 and 1, while *μ* denotes the step size. We adopt a specific definition of fractional derivatives (FD) known as the generalized Euler Gamma function. This definition of chain rule incorporates an engineering approximation. This approximation is simple to implement, as $f(t)=x^{n}$:

$$D^{\beta }f(t)=\frac{\partial^{\beta }f(t)}{\partial^{\beta }h}=\frac{\Gamma (m+1)}{\Gamma (m-\beta +1)}x^{n-\beta }$$ (40)

Here $D^{\beta }=\frac{\partial^{\beta }}{\partial^{\beta }h}$ is a fractional operator and *Γ* a Gamma function:

$$\Gamma (z)=\int_{0}^{\infty }t^{z-1}e^{-t}dt$$ (41)

By fractional order differentiation of Eq (36) with respect to ***h*** yields:

$$\frac{\partial^{\beta }}{\partial^{\beta }h}J(t)=-2(e(t)x(t-k))D^{\beta }h(t)$$ (42)

The following fractional correcting term is obtained by employing Eqs (40) and (42):

$$\frac{\partial^{\beta }}{\partial^{\beta }h}J(t)=-2(e(t)x(t-k))\frac{1}{\Gamma (2-\beta )}x^{1-\beta }$$ (43)

First order differentiation of Eq (36) with respect to ***h*** results in:

$$\frac{\partial J(t)}{\partial h}=-2e(t)x(t-k)$$ (44)

By putting Eqs (43) and (44) in Eq (39), it provides a weight adaptation equation for the FLMS:

$$h(t+1)=h(t)+\mu e(t)x(t)+\mu e(t)x(t)\frac{1}{\Gamma (2-\beta )}h^{1-\beta }$$ (45)

Its vector representation is given below:

$$h(t+1)=h(t)+\mu e(t)x(t)+\mu e(t)x(t)⊙\frac{1}{\Gamma (2-\beta )}h^{1-\beta }$$ (46)

Here ⨀ is the operator for the multiplication of vectors element wise.

### Convergence analysis of FLMS

To know the FLMS convergence properties, some other frame of reference is used. The *u*(*t*) is defined $u(t)=h(t)-h_{\text{o}}$, the optimal weight ***h***_0_ cancels out the effects of interferers, and due to these weights error produced:

$$e_{o}(t)=d(t)-x^{T}(t)h_{o}$$ (47)

Weight vector *u*(*t*) at instant (*t* + 1) is expressed as:

$$u(t+1)=u(t)+\mu_{1}e(t)x(t)+\mu_{2}e(t)x(t)⊙\frac{1}{\Gamma (2-\beta )}u{\left(t\right)}^{1-\beta }$$ (48)

For keeping the things simple and prevent the creation of complex numbers, put $\mu_{2}=\Gamma (2-\beta )\mu_{1}$ and absolute value of the fractional part of weight is selected. Expectation is applied to the two sides for statistical independence, and the relationship is obtained by using the value of *e*_*o*_(*t*).


$$E[u(t+1)]=\left[I-\mu_{1}R+\mu_{1}R⊙E\left[|u(t){|}^{1-\beta }\right]\right]E[u(t)]$$ (49)

When further simplified the equation turns to:

$$E[u(t+1)]=\left[I-\mu_{1}R\left(I-I⊙E\left[|u(t){|}^{1-\beta }\right]\right)\right]E[u(t)]$$ (50)

Replacing $I⊙E\left[|u(t){|}^{1-\beta }\right]\;by\;F(h(t),\beta )$ the relation becomes:

$$E[u(t+1)]=\left[I-\mu_{1}R(I-F(h(t),\beta )]E[u(t)]\right.$$ (51)

With the progression of iterations, the anticipated trend in the mean power of the weight difference is expected to exhibit a decreasing pattern. We conclude, in a steady state that:

$$\underset{t\to \infty }{lim}∥E[u(t)]{∥}^{2}=0$$ (52)

The condition is true only for $-1<I-\mu_{1}R(I-F(h(t),\beta )<1$, and upon performing simplifications, the expression can be formulated as $I<\mu_{1}<\frac{2}{R-F(h(t),\beta )}$. The relationship can alternatively be expressed with reference to the maximum eigenvalues *λ*_*max*_ as follows:

$$I<\mu_{1}<\frac{2}{\lambda_{max}-F(h(t),\beta )}$$ (53)

Eq (53) offers a choice of taking of step sizes for fractional algorithm convergence. This equation introduces an extra non-linear component resulting from the fractional processing, which in turn simplifies the process of fine-tuning the correlation matrix. What’s particularly valuable is that this approach ensures improved convergence, even when employing larger step sizes.

## Results and discussion

**Fig 3:**
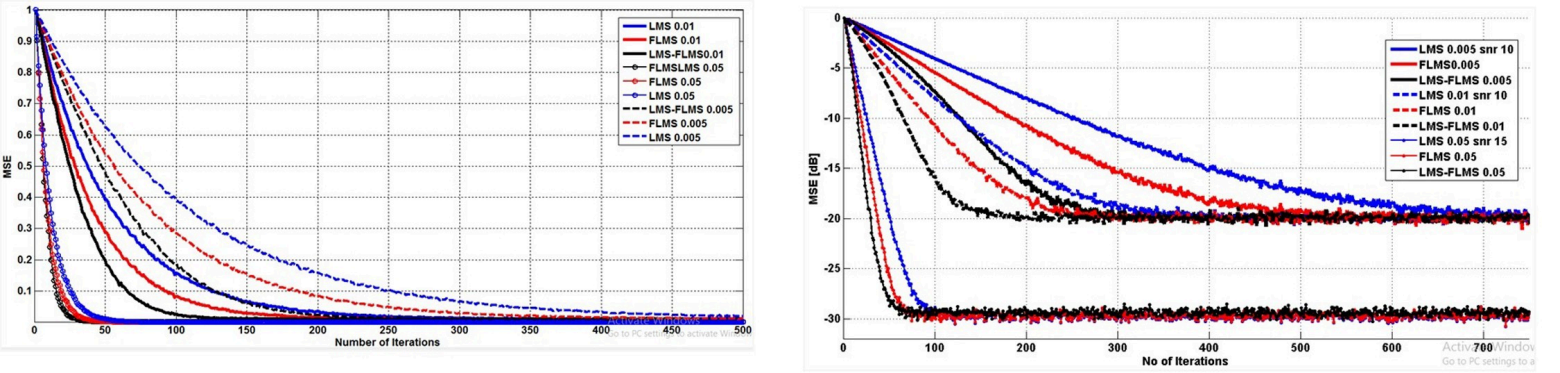
**a.** Convergence performance. **b.** MSE performance.

**Fig 4:**
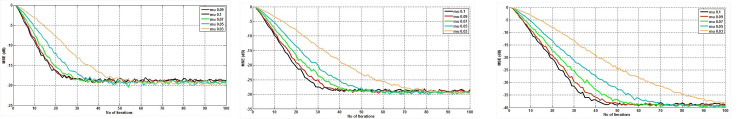
**a.** MSE learning curves of the LFLMS with a fractional value of 0.9 and SNR of 10dB. **b.** MSE learning curves of the LFLMS with a fractional value of 0.9 and SNR of 15dB. **c.** MSE learning curves of the LFLMS with a fractional value of 0.9 and SNR of 20dB.

**Fig 5:**
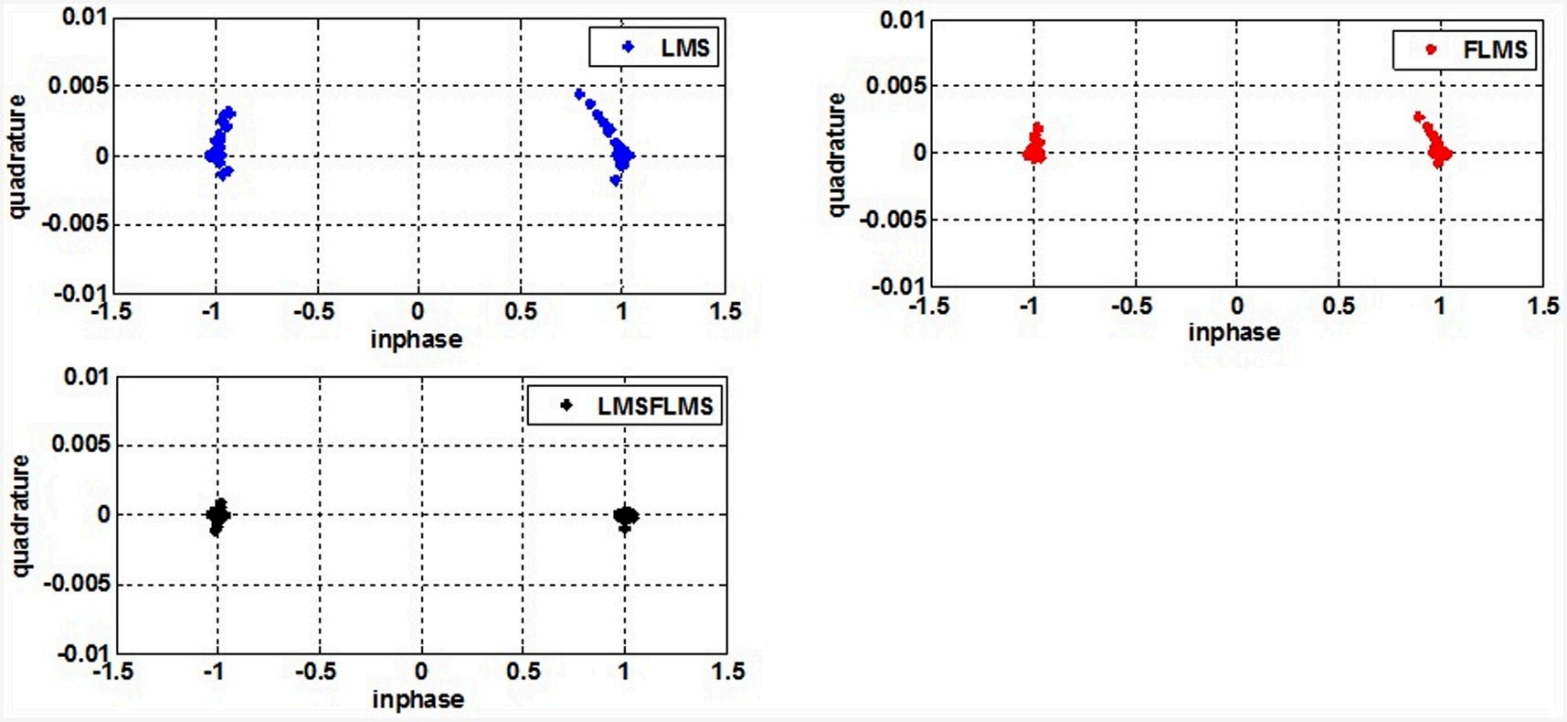
Comparison of scatter diagrams for the LMS, FLMS, and LFLMS.

**Fig 6:**
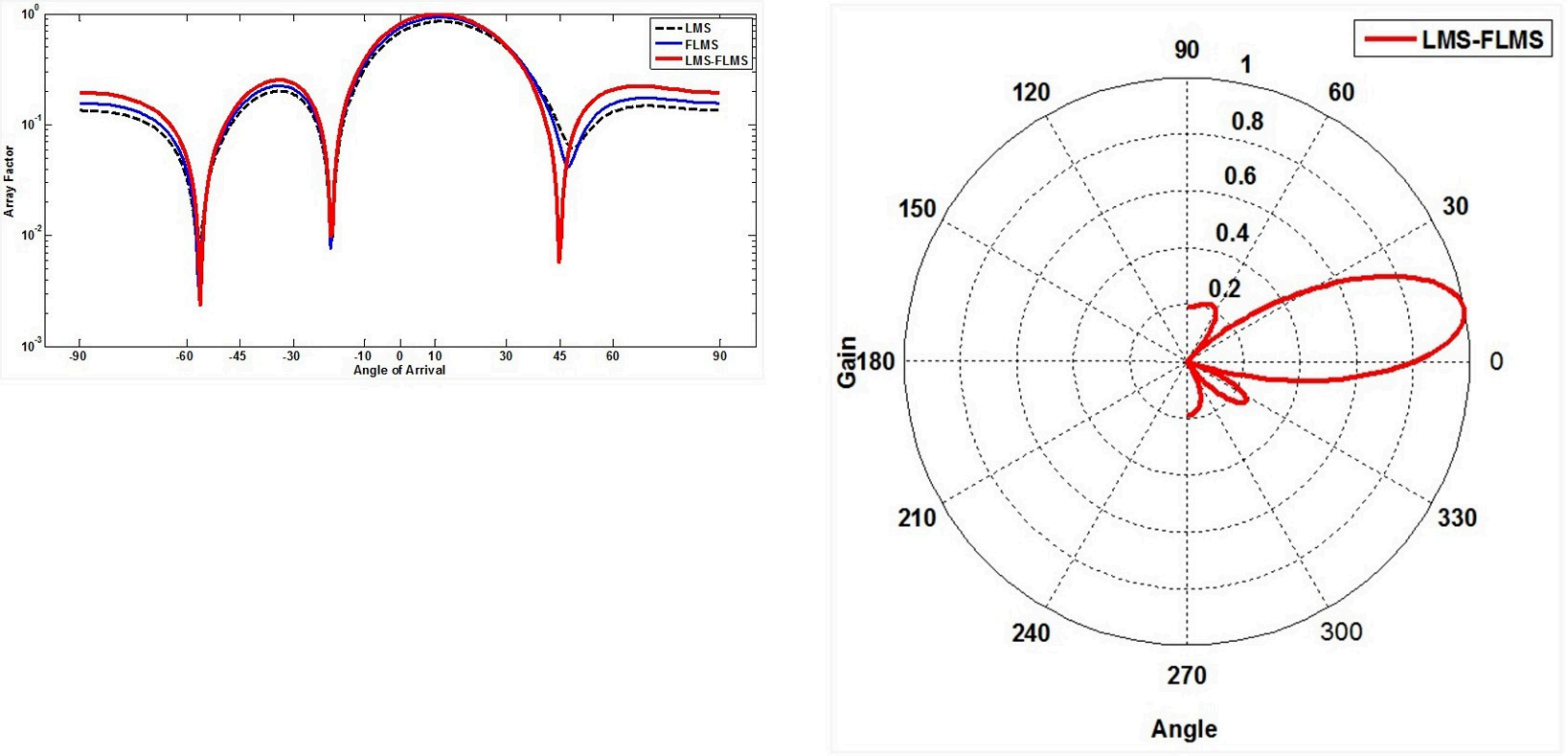
**a**. Beam pattern SNR = 15, *β* = 0.9, *μ* = 0.01. **b.** Polar beam pattern for SNR = 15dB, ***β* = 0.9**, and *μ* = 0.01

The proposed fractional self-correcting approach was evaluated by simulations with Monte Carlo. Throughout, a comprehensive analysis was conducted to observe how the algorithm performed across various conditions of SNR, sizes, and fractional values, investigating its behavior and adaptability in diverse scenarios. Specifically, the simulations compared the convergence speeds and mean squared error plots for 500 independent runs and 1000 samples. There are 20 isotropic array elements of ULA, equally spaced (*d* = 0.5λ). The interferer and intended signal arrive at 45° and 10°, accordingly. This research utilizes defined AOA. Augmenting this research with diverse AOA estimation methodologies allows for comprehensive examination and comparison of outcomes, enriching the investigation of AoA estimation and tracking techniques. Table 2 summarizes the parameters applied in these simulations. The outcomes are presented in various figures, comparing the suggested LFLMS algorithm with the fractional LMS and its traditional counterpart. Fig 3A and 3B compare the efficiency of convergence and MSE performance of the recommended and other schemes for various parameters. Fig 4A, 4B and 4C contrast the MSE plots of LFLMS for various parameters. Fig 5 presents a comparative analysis through a scatter plot, highlighting the performance differences among LFLMS, FLMS, and LMS. Finally, Fig 6A and 6B illustrate and compare the beampattern performance exhibited by the LFLMS, FLMS, and LMS techniques. These results offer valuable understanding regarding the algorithm’s functionality and showcase its efficacy across diverse conditions.

**Table 2:** Values of the parameters employed.

Parameter	Value
Array size	4, 20
Samples	1000
Runs	500
Step sizes	0.5, 0.1, 0.09, 0.05
SNR	10,15,20
DOA of desired Signal	10°
DOA of interferer signal	45°

### MSE and convergence performance

Fig 3A illustrates the clear superiority of LFLMS in convergence speed over both FLMS and LMS across different SNR values. Specifically, with SNR values of 10dB and 20dB, step sizes of 0.1, 0.05, and 0.005, and a fractional value of 0.9, the proposed LFLMS achieves convergence in 25 iterations, whereas FLMS and LMS require 35 and 50 iterations, correspondingly. It shows that LFLMS achieves convergence rates that are 50% faster than LMS and 29% faster than FLMS. Similarly, for a step size of 0.01 and an SNR of 10dB and LFLMS achieve convergence in 105 successive instances, compared to 248 iterations for LMS and 180 iterations for FLMS. LFLMS demonstrates 57% and 42% faster convergence compared to traditional and fractional LMS, accordingly. The same way, for an SNR value of 10dB and a step size of 0.005, LFLMS requires 220 instances to converge, while LMS and FLMS require 531 and 388 instances, respectively. In this scenario, its convergence performance is 59% quicker than LMS and 43% faster than FLMS. Fig 3A displays plots for different SNR values, while Tables 3 and 4 summarize the convergence behavior, offering statistical data for clearer comparison.

**Table 3:** Convergence comparisons of LMS and LFLMS for different step sizes and SNR values.

Step Size	SNR	No of Iterations of Convergence		LFLMS Convergence Improvement over LMS
		LMS	LFLMS	
0.05	10	50	25	50%
0.05	20	48	23	47%
0.01	10	248	105	57%
0.01	20	273	119	56%
0.005	10	531	220	59%
0.005	20	525	226	57%

**Table 4:** Convergence comparisons of FLMS and LFLMS for different step sizes and SNR values.

Step Size	SNR	No of Iterations of Convergence		LFLMS Convergence Improvement over FLMS
		FLMS	LFLMS	
0.05	10	35	25	29%
0.05	20	33	23	30%
0.01	10	180	105	42%
0.01	20	199	119	40%
0.005	10	388	220	43%
0.005	20	379	226	40%

Fig 3B presents a comparison of the algorithms across various step sizes and SNR values. The plots depict MSE comparisons at specific iterations. For instance, with an SNR value of 10dB, a step size of 0.005, and a fractional order of 0.9, at the 250th iteration, conventional LMS, fractional LMS, and LFLMS converge to MSE values of -10.17dB, -13.57dB, and -18.85dB respectively. This indicates a LFLMS MSE improvement of 8.68dB or 46% over LMS and 5.28dB or 28% over FLMS.

Tables 5 and 6 offer a statistical breakdown of the plots to facilitate comparison across various values. Specifically, for 10dB SNR and a step size of 0.01, at the 100th iteration, the proposed LFLMS exhibits a significant MSE improvement of 7.79dB or 49% compared to the LMS. Additionally, LFLMS demonstrates a 32% improvement over FLMS for identical parameters.

**Table 5:** MSE performance comparison of LMS and LFLMS for different step sizes and SNR values.

Step Size	SNR	Iteration of Convergence	MSE values(dB)		LFLMS MSE Improvement over LMS
			LMS	LFLMS	
0.005	10	250	-10.17	-18.85	46%
0.005	10	350	-13.74	-20.11	32%
0.01	10	100	-7.97	-15.76	49%
0.01	10	150	-11.59	-18.97	39%
0.05	20	40	-16.02	-27.31	41%
0.05	20	60	-23.8	-36.37	35%

**Table 6:** MSE performance comparison of FLMS and LFLMS for different step sizes and SNR values.

Step Size	SNR	Iteration of Convergence	MSE values(dB)		LFLMS MSE Improvement over FLMS
			FLMS	LFLMS	
0.005	10	250	-13.57	-18.85	28%
0.005	10	350	-17.38	-20.11	14%
0.01	10	100	-10.68	-15.76	32%
0.01	10	150	-15.01	-18.97	21%
0.05	20	40	-21.91	-27.31	20%
0.05	20	60	-32	-36.37	12%

### MSE behaviour against varying step sizes

Fig 4A, 4B and 4C illustrate that reducing the step size while keeping the SNR and fractional order constant results in a diminished convergence rate. Particularly, under conditions of an SNR of 10dB and a fractional value of 0.9, LFLMS reaches a convergence of the MSE value of -15dB in 18 iterations with a step size of 0.1, whereas with a step size of 0.03, convergence occurs by the 36th iteration. The LFLMS performs better in steady-state for smaller step sizes, as demonstrated in Fig 4A. Likewise, in Fig 4B, a similar trend is observed with varying step sizes at an SNR of 15dB. In Fig 4C, when analyzing the graph for 20dB SNR, it becomes apparent that augmenting step size improves the rate at which convergence occurs.

### Scattered plots performance

The scatter plot presents a comparison between LFLMS, FLMS, and LMS using an SNR of 20dB, *β* = 0.9, and *μ* = 0.1. Results from Fig 5 suggest that, in the context of scatter plots, LFLMS outperforms both the fractional and conventional LMS. Notably, the following plot depicting the proposed LFLMS showcases data with superior accuracy and precision in comparison to those observed in FLMS and LMS. These findings highlight the efficacy of the LFLMS algorithm in this experimental context, demonstrating its ability to yield more accurate and focused results amidst varying conditions, as depicted by the visual representation in Fig 5.

### Beampatterns

Fig 6A and 6B illustrate the precision in steering the beam towards the angle of arrival (10°) of the desired signal while cancelling out the interfering signal (45°) through beampatterns. To highlight distinctions clearly, these plots use four antenna elements, showcasing that reducing the number of elements widens the lobes. In Fig 6A, representing an SNR of 15dB, a step size of 0.01, and a fractional value of 0.9, the LFLMS accurately directs the main beam at 10°, outperforming the FLMS and LMS.

Similarly, in Fig 6B, for the same parameters, the LFLMS demonstrates superior directivity in the polar plot results.

## Conclusion

This study introduces the LFLMS algorithm, a fractional order approach designed to enhance modern communication systems, i.e., Internet of Things (IoT) and radar systems. By incorporating fractional derivatives, it minimizes eigenvalue spreading in the autocorrelation matrix, thereby improving convergence and steady-state response. Simulation results indicate LFLMS achieves 59% and 43% faster convergence compared to fractional LMS and LMS, with 49% and 32% MSE improvement relative to LMS and FLMS. Furthermore, it enhances directivity and gain, benefiting signal coverage and quality. The LFLMS algorithm finds versatile applications, extending its utility across various domains, including contemporary engineering disciplines such as control, instrumentation, and the evolving landscape of IoT-based communication systems in future generations. The study encourages further research exploring LFLMS algorithm performance by varying parameters, like array elements, interferers, and modulation schemes. Such investigations can yield valuable insights into enhancing adaptive beamforming algorithms. As future work, we intend to augment this investigation by incorporating AOA estimation methodologies employing a diverse array of algorithms. This study lays a solid groundwork for exploring fractional order adaptive algorithms’ potential in diverse signal processing applications i.e. system identification, echo noise control, and bio medical engineering etc.

## Supporting information

S1 Dataset (MAT)

S2 Dataset (MAT)
